# Primary Pancreatic Lymphoma Masquerading as Carcinoma

**DOI:** 10.1155/2020/5160545

**Published:** 2020-02-26

**Authors:** Nasser A. N. Alzerwi

**Affiliations:** Department of Surgery, College of Medicine, Majmaah University, Ministry of Education, Al-Majmaah City, 11952, P. O. Box 66, Riyadh Region, Saudi Arabia

## Abstract

Adenocarcinoma is the most common primary pancreatic neoplasm type, followed by secondary pancreatic lymphoma and primary pancreatic lymphoma (PPL). PPL is associated with peripancreatic lymphadenopathy and usually presents as a homogenous mass with extrapancreatic invasion into surrounding structures. However, localized involvement of the distal pancreas is uncommon, and diffuse involvement of the pancreas is even rarer. Herein, we present the case of a 53-year-old woman with PPL of the uncinate process with biliary obstruction mimicking pancreatic adenocarcinoma, successfully diagnosed nonoperatively. Abdominal computed tomography showed an ill-defined uncinate process mass, hypodense with mild enhancement (3.9 × 3.4 × 3.5 cm), infiltrating the second and third parts of the duodenum. Biopsy revealed NHL with no evidence of adenocarcinoma. PPL was diagnosed. She received chemotherapy with a CHOP (cyclophosphamide, doxorubicin, vincristine, and prednisone) protocol with rituximab, which she tolerated with no clinical or radiological evidence of recurrence at 1-year follow-up.

## 1. Introduction

Primary pancreatic lymphoma (PPL) accounts for approximately 0.5% of pancreatic neoplasms and <2% of lymphomas [1, 2] and commonly presents as non-Hodgkin lymphoma [3, 4]. Its clinical manifestations and radiological findings mimic those of other pancreatic lesions such as adenocarcinoma, neuroendocrine tumors, and chronic pancreatitis [2]. The rarity, difficulty in access, dissimilar outcomes, treatment, and survival rates of PPL make it a diagnostic and therapeutic challenge [3, 4]. Histological diagnosis is essential [5] as histological subtype is a significant prognostic factor [1, 2, 6, 7]. Herein, we present the case of a 53-year-old woman with PPL of the uncinate process with biliary obstruction mimicking pancreatic adenocarcinoma and review the literature on PPL.

## 2. Case Presentation

A 53-year-old woman presented to the emergency department complaining of intermittent sharp epigastric pain radiating to the back, associated with nausea, scleral and cutaneous icterus, itching, dark urine, pale stool, and early satiety. She had an unremarkable medical and family history. Systemic examination revealed no other symptoms.

Upon examination, the patient looked cachectic, conscious, and alert but dehydrated and deeply jaundiced. Her vital signs were within normal limits; she was afebrile. Abdominal examination revealed no scars or prominent veins; the abdomen was soft and lax with mild tenderness at the epigastrium, with no palpable mass, organomegaly, or lymphadenopathy. Normal bowel sounds were audible, with intact hernial orifices. A digital rectal exam showed unremarkable results. Laboratory tests revealed direct hyperbilirubinemia (Table 1) and anemia.

The clinical impression was obstructive jaundice requiring further investigation. She was admitted and prescribed intravenous fluid therapy.

**Figure 1:**
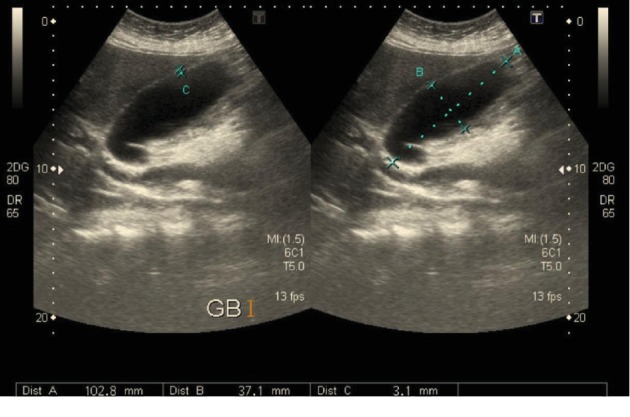
Transabdominal ultrasonography showing a distended gall bladder (102.8 × 37.1 mm) with a thin wall (3.1 mm) and tortuous dilated cystic duct with no intraluminal gallstones or pericholecystic fluid.

**Figure 2:**
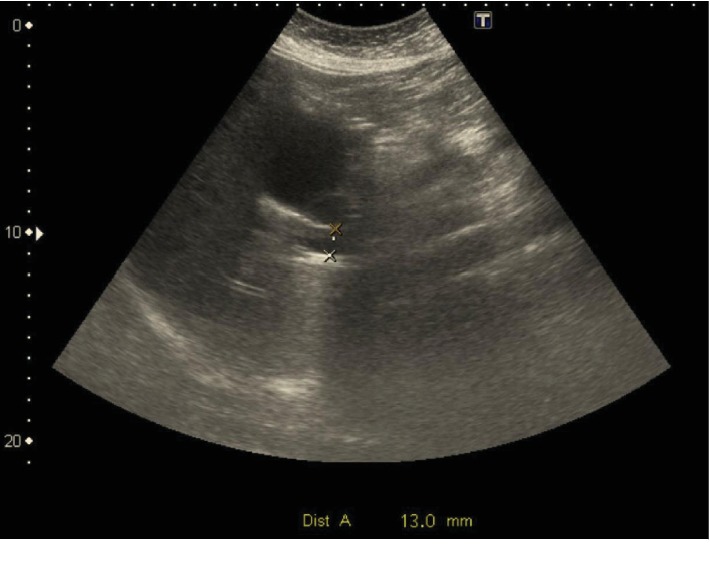
Transabdominal ultrasonography showing a dilated common bile duct (13 mm) with no visible choledocholithiasis.

Chest X-ray showed unremarkable results; abdominal ultrasonography (US) revealed distended gallbladder with no stones or signs of acute cholecystitis (Courvoisier's sign). However, a tortuous cystic duct and dilatation of the intrahepatic and extrahepatic biliary ducts and the common bile duct (CBD) (13 mm) (Figures 1 and 2) were observed.

**Figure 3:**
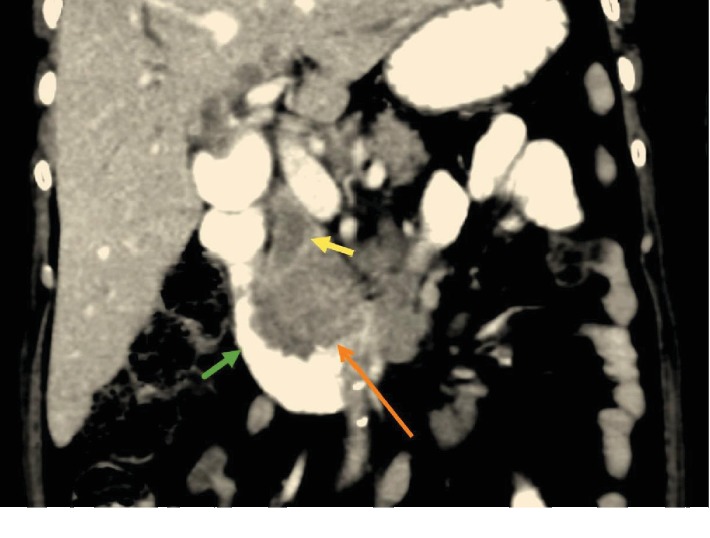
Enhanced computer tomography (CT) of the abdomen. Coronal slice obtained through the second part of the duodenum (D2; green arrow) shows an uncinate process mass (orange arrow) protruding into the lumen of D2, infiltrating and obstructing the dilated distal common bile duct (yellow arrow).

Abdominal computed tomography (CT), performed for suspected obstructive jaundice due to malignancy, showed findings similar to US findings and an ill-defined uncinate process mass that was hypodense with mild enhancement (3.9 × 3.4 × 3.5 cm). The mass had infiltrated the second and third parts of the duodenum with no signs of duodenal obstruction and mass effect on the inferior vena cava (IVC). There was extensive lymphadenopathy (perilesional, retroperitoneal, mesenteric, and left para-aortic) with no metastatic deposits in the liver (Figure 3).

**Figure 4:**
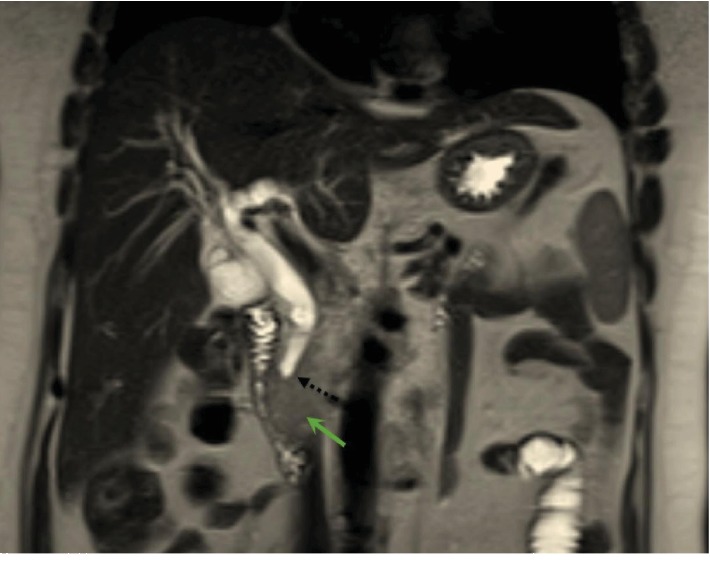
Magnetic resonance imaging (MRI) of the abdomen. Coronal slice obtained through the second part of the duodenum (D2) shows the uncinate process mass (green arrow) obstructing the distal common bile duct (dotted black arrow).

**Figure 5:**
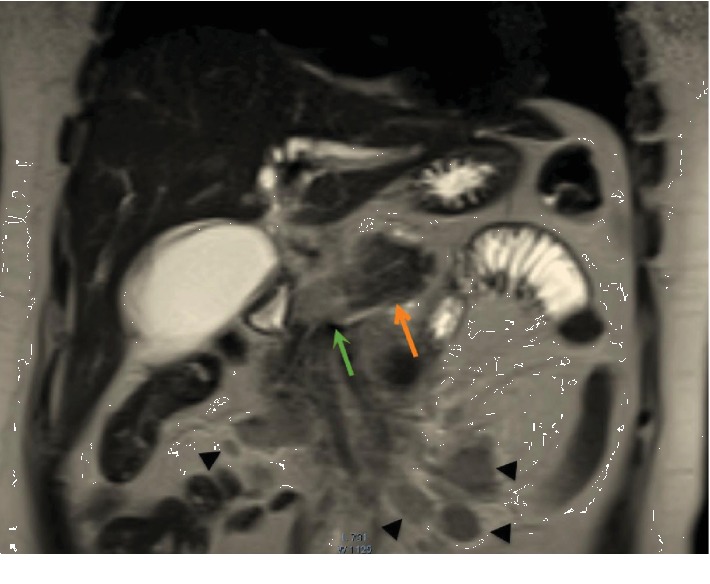
Magnetic resonance imaging (MRI) of the abdomen. Coronal slice shows the line of demarcation between the distal normal body and tail of the pancreas (orange arrow) and the neoplastic proximal body (green arrow), with multiple mesenteric and peripancreatic neoplastic lymph nodes (black arrowheads).

**Figure 6:**
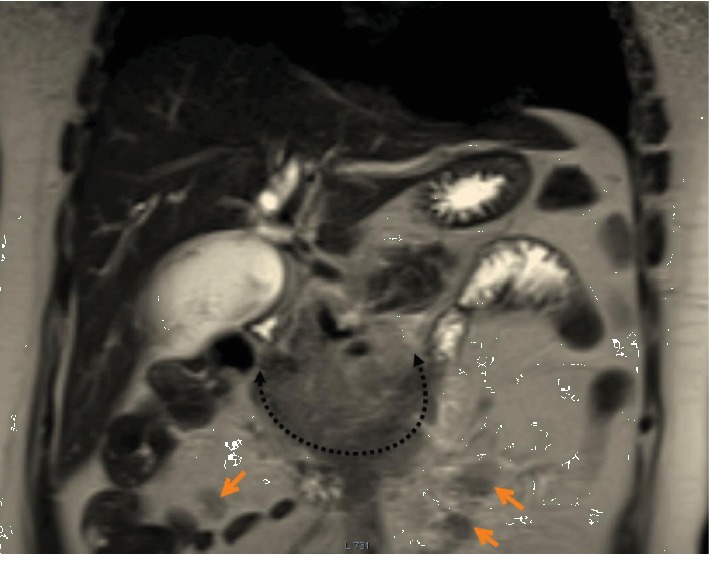
Magnetic resonance imaging (MRI) of the abdomen. Coronal slice shows the uncinate process mass (dotted black arrow) and multiple neoplastic peripancreatic lymph nodes (orange arrows).

**Figure 7:**
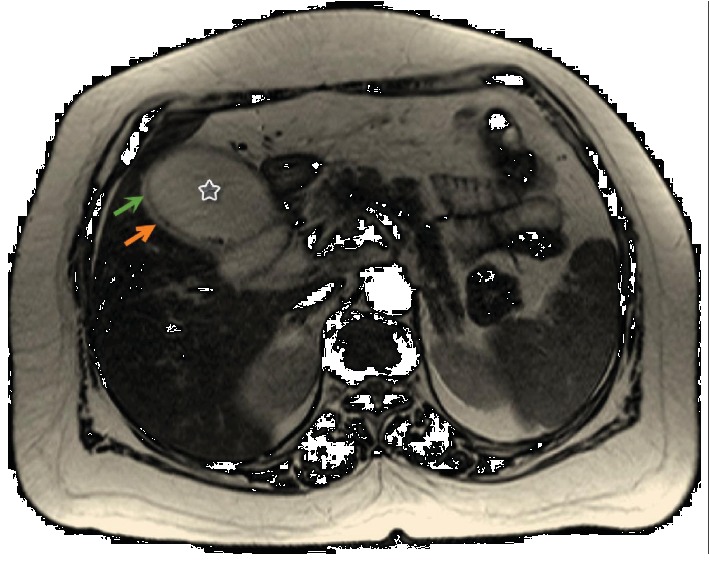
Magnetic resonance imaging (MRI) of the abdomen. Axial slice obtained through the distended GB (star) shows the thin gall bladder wall (green arrow) and a thin rim of pericholecystic fluid (orange arrow).

Chest CT showed no metastatic lung deposits or mediastinal lymphadenopathy. Tumor markers were within the normal range (Table 1). Magnetic resonance cholangiopancreatography (MRCP) showed diffuse intrahepatic biliary duct dilatation, a dilated CBD (13 mm), and a normal pancreatic duct (Figures 4–7).

**Figure 8:**
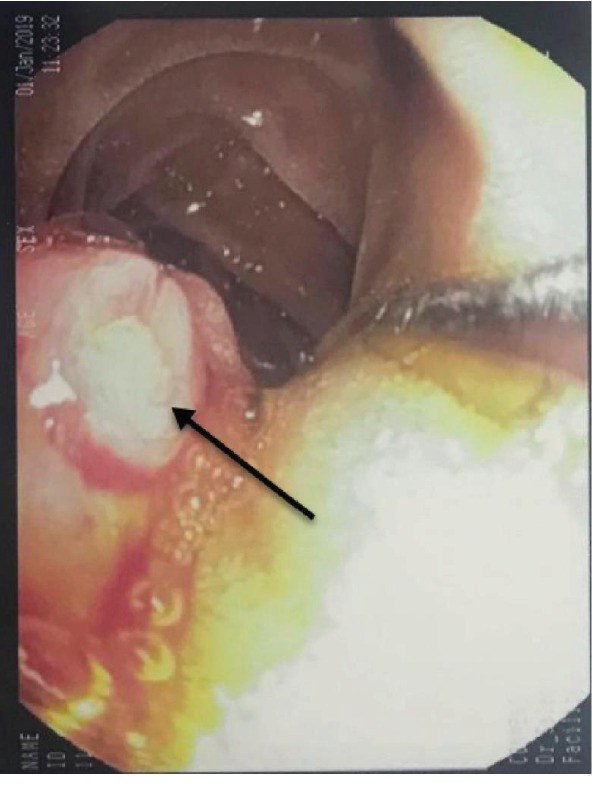
Duodenoscopy shows stenotic major papilla with neoplastic overgrowth (black arrow) protruding into the lumen of the second part of the duodenum (D2).

**Figure 9:**
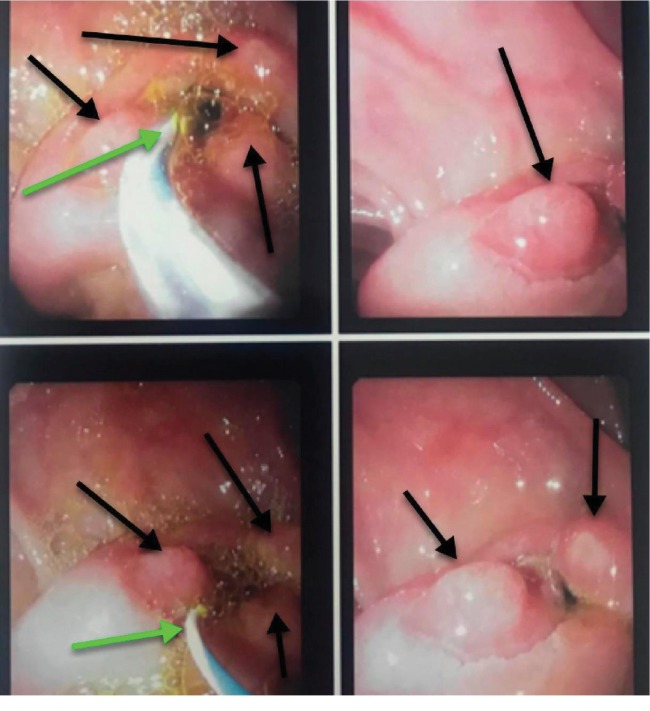
Duodenoscopy shows the cannulation of the stenotic major papilla (green arrows) and the granular appearance of the neoplastic mass (black arrows).

**Figure 10:**
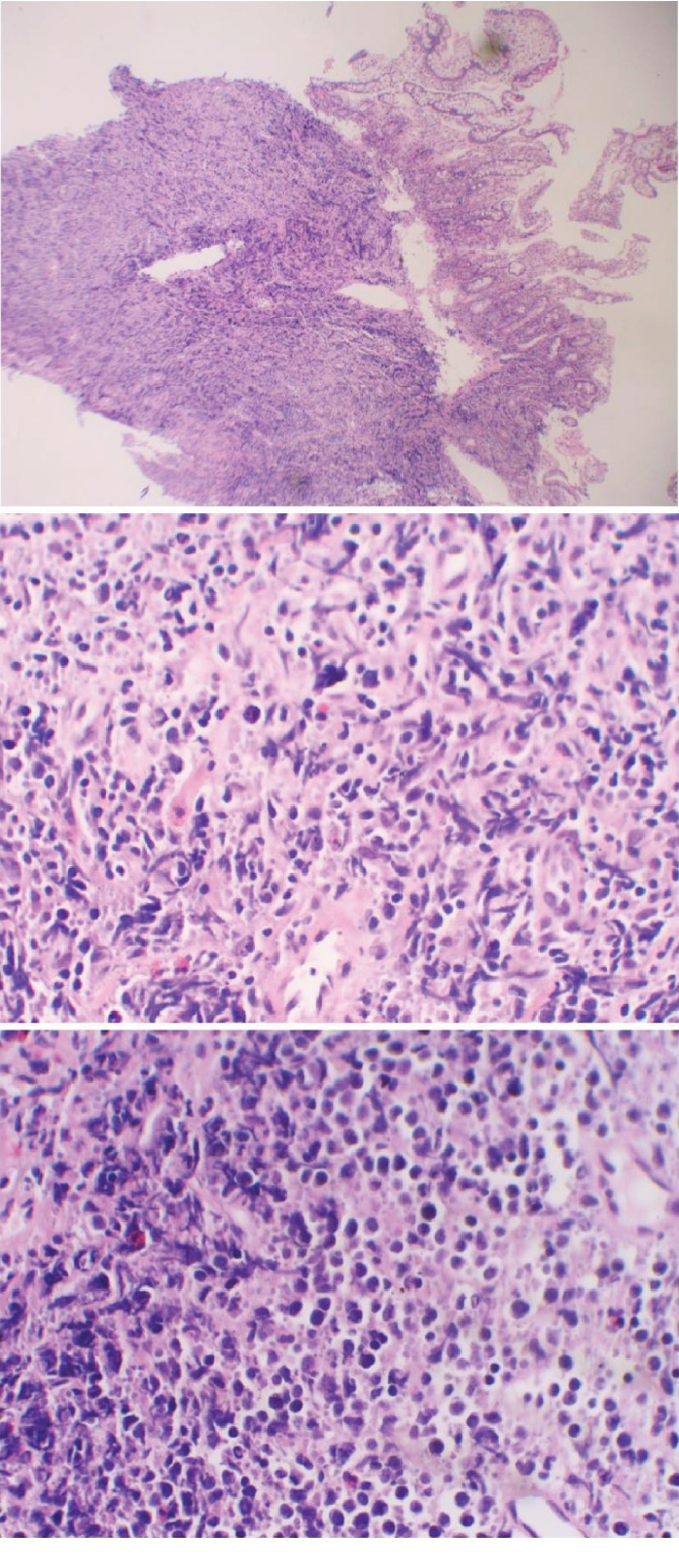
Cross-sectional biopsy shows an infiltrate of large atypical pleomorphic lymphoid cells, consistent with large diffuse B-cell lymphoma (hematoxylin-eosin).

Duodenoscopy showed mucosal erythema on stenotic and granular major papilla in the periampullary area; thus, a biopsy was performed (Figure 8). Endoscopic retrograde cholangiopancreatography (ERCP) showed distal CBD and papillary stenosis, which required sphincterotomy and stenting, and an ampullary mass, which was biopsied (Figure 9). Biopsy revealed NHL with no evidence of adenocarcinoma (Figure 10). Whole-body multislice CT was negative for generalized lymphadenopathy, and the multidisciplinary oncology board diagnosed PPL. She received cyclophosphamide, doxorubicin, vincristine, and prednisone (CHOP) with rituximab with good tolerability, and there were no clinical or radiological evidence of recurrence after 1 year of follow-up. Written informed consent was obtained from the patient for the publication of this report. The institutional review board of King Fahad Medical City (Riyadh, KSA, Tel: +96612889999 ext. 26913, email: okasule@kfmc.med.sa) waived the need for ethical approval on December 05, 2019 (IRB log number: 19-613E).

## 3. Discussion

The typical age of PPL patients is 35–75 years (mean: 55 years), with a strong male predilection (male : female ratio of 7 : 1) [8]. PPL presents with typical symptoms of lymphoma like fever, chills, and night sweats in only 2% of cases [8]. The clinical presentations of PPL are nonspecific, most commonly abdominal pain followed by abdominal mass detection, weight loss, jaundice (76%), and acute pancreatitis (AP) (18%), which mimics adenocarcinoma. Though PPL is rare, due to the differences in the prognosis and treatment approaches of adenocarcinoma and PPL (surgical resection for adenocarcinoma, while chemotherapy for PPL), it is critical to differentiate PPL from adenocarcinoma [1].

PPL is most commonly located at the head of the pancreas, though it can be found in the body or tail. The tumor size ranges from 4 to 17 cm on radiological scans [9].

Imaging plays a crucial role in pancreatic mass diagnosis [1]. CT is commonly used in the detection and characterization of PPLs appearing as homogenous masses with less contrast enhancement compared with pancreatic adenocarcinomas [1].

There are two different morphological patterns of PPL: localized, well-circumscribed tumors and diffuse tumors infiltrating or replacing most of the pancreas [1, 10].

The radiological criteria for differentiating PPL from more common pancreatic adenocarcinomas are a bulky localized tumor in the pancreatic head, no significant dilatation of the pancreatic duct, enlarged lymph nodes below the renal vein level, invasive tumor growth, and retroperitoneal or upper abdominal organ infiltration [1]. All of these criteria were fulfilled in our case.

However, the definitive diagnosis of PPL is impossible from imaging alone, and pathologic examination (cytohistopathological confirmation) is imperative [1]. Immunohistochemical staining and flow cytometry are essential as hematoxylin-eosin staining findings alone may resemble those of a poorly differentiated carcinoma, limiting the value of fine needle aspiration cytology (FNAC) as a diagnostic technique [11–14].

The serum CA 19-9 level in PPL patients is usually normal or sometimes slightly elevated in the case of biliary obstruction [15]. In our case, the CA 19-9 level was normal despite the clinical, biochemical, and radiological evidence of biliary obstruction.

The management and prognosis of PPL depend on the tumor stage and grade. The most common subtype of PPL is the diffuse large B-cell subtype, and long-term remission has been obtained with chemotherapy (CHOP); in the case of CD20-positive diffuse large B-cell lymphoma, the addition of rituximab increases the remission rate [3, 15, 16].

PPL patients treated with chemotherapy alone have a better prognosis than do pancreatic adenocarcinoma patients. PPL patients have a 5-year survival rate of 26%–66% [3, 15]. The therapeutic role of radiotherapy in PPL is not yet well defined [3]. Multiple studies have shown a therapeutic advantage of adjuvant radiotherapy when combined with chemotherapy for NHL; however, the replication of such superior outcomes in PPL is not as well established [17–19]. Few case series studies have shown promising results, Bouvet et al. recommended the routine use of involved-field radiotherapy after chemotherapy in a survival report of 9 out of 11 patients at a median follow-up time of 67 months [20]. Behrns et al. reported a mean survival of 13 months, 22 months, and 26 months, for those who received chemotherapy alone, radiotherapy alone, and combined chemoradiotherapy, respectively [21]. The superior outcome of combined chemoradiotherapy could be due to the superior local control effect of radiotherapy, as most therapeutic failures were local rather than systemic [22, 23].

PPL should be considered in the differential diagnosis of pancreatic head masses, especially when there is extensive lymphadenopathy localized to the peripancreatic region, and should be ruled out with transluminal biopsy to avoid unnecessary surgical intervention. However, if adenocarcinoma cannot be safely ruled out, open biopsy with frozen section should be considered before performing pancreaticoduodenectomy (PD), as most cases are diagnosed post-PD. Furthermore, the more common variant (secondary pancreatic lymphoma) should be ruled out before diagnosing PPL.

## Figures and Tables

**Table 1:** Laboratory results.

Test	Result	Units
WBC	5.9 × 10^9^	Cells/L
Hgb	9.7	g/dL
Platelets	220 × 10^9^	/L
INR	0.9	—
PT	10.4	sec
PTT	39.4	sec
AST	146	U/L
ALT	230	U/L
Alp	358	U/L
TB	159.8	*μ*mol/L
DB	139.2	*μ*mol/L
Alb	33.4	g/L
Amylase	22	U/L
GGT	466.8	U/L
Urea	3.4	mmol/L
Creatinine	60.7	*μ*mol/L
K	3.8	mmol/L
Na	139	mmol/L
LDH	128	IU/L
CEA	0.8	ng/mL
CA 19-9	16	U/mL
*AFP*	*2.1*	*ng/mL*

WBC: white cell count; Hgb: hemoglobin; INR: international normalized ratio; PT: prothrombin time; PTT: partial thromboplastin time; AST: aspartate aminotransferase; ALT: alanine aminotransferase; Alp: alkaline phosphatase; TB: total bilirubin; DB: direct bilirubin; Alb: albumin; GGT: gamma glutamyl transferase; K: potassium; Na: sodium; LDH: lactate dehydrogenase; CEA: carcinoembryonic antigen; CA 19-9: carbohydrate antigen 19-9; AFP: alpha fetoprotein; L: liter; g: gram; dL: deciliter; sec: seconds; U: unit; mmol: millimole; *μ*mol: micromole; IU: international unit; ng: nanogram; mL: milliliter.
